# The Civic Hospital of Venice

**Published:** 1908-07-04

**Authors:** Conrad W. Thies


### HOSPITAL ADMINISTRATION.

### CONSTRUCTION AND ECONOMICS.

### THE CIVIC HOSPITAL OF VENICE.

By CONRAD W. THIES.

The City of Venice, which for over a thousand years held sway over a considerable part of Northern Italy and the coasts of the Adriatic, was not only great in wealth, commerce, and the Arts, but was also munificent in its charities. From remote times the city founded many religious communities, which had for one of their objects the amelioration of the condition of the poorer classes: the early records tell of numerous asylums for foundlings and orphans, also of many hospices, or hospitals, for the sick and suffering. Some of these latter institutions were specially founded for the reception of the large numbers of pilgrims and crusaders who passed through the city on their journeys to and from the Holy Land; Venice at that period being the principal port of departure from Europe for the East.

In the year 1346 Peter of Assisi founded a hospital for lepers on the island of San Lazzaro, and at the close of the sixteenth century this hospital was transferred to the city, and, after many changes, has gradually been developed into the present "Ospedale Civile" or "Civic Hospital of the City of Venice." This great hospital is situated on the Campo, or square, where stands the well-known monument to Bartholomeo Colleoni, which is considered to be the finest equestrian statue in the world. The present buildings are of various ages and styles of architecture; the southern portion was originally the College of San Marco, and was built by the brothers Lombardi in the fifteenth century. Ruskin has expressed his opinion that this building is one of the most perfect specimens of the architecture of that period now left in Venice. The beautiful lunette over the entrance gateway, and the two fine relievos—"The healing of the cobbler Anianus" and "St. Mark baptising a convert"—are well worthy of attention. Another portion of the hospital was formerly the monastery of an order of preaching friars, and was erected in the thirteenth century, while other parts are quite modern and were built for the special purposes of the hospital.

After passing through numerous vicissitudes, owing to the many changes of Government which Venice has experienced during the past 150 years, the hospital and all the other charitable institutions of the city were in 1879 placed by the Government of United Italy under the administration of the "Congregazione di Carita"; but in the following year a special charter was granted, and the management of the hospital was thereby entrusted to a "Council of Administration," the members of which were jointly nominated by the Government and the city authorities.

The hospital now contains 1,200 beds, of which over 1,000 are in daily use. The patients admitted number about 13,000 per annum, or about 8 per cent. of the whole population of Venice. Upon entering the hospital it is at once evident that many of the buildings were originally designed for other purposes than for the treatment of the sick. The staircases and passages are of a distinctly ecclesiastical character, and this impression is accentuated upon entering some of the larger wards, which are in fact great halls or chapels.

It is a novel experience to enter the largest of these wards, which is about 150 feet long by 50 feet in width, and provides accommodation for about 100 beds, which are placed in long rows, without any screens or divisions of any kind; the entire end of this great hall is occupied by a raised marble altar.

The admission to the hospital is free to the poor of the city, but no chronic cases are admitted. In all cases the applicant for admission must furnish a certificate of indigence, which must be accompanied by a declaration by a medical man that the person is a fit subject for hospital treatment and cannot be properly tended at home. It is considered by the authorities to be to the advantage of the community that, as far as is practicable, the sick shall be treated in their own homes, as the bonds of family life are thus strengthened. Patients are only admitted from districts outside Venice in exceptional cases, when there is room in the hospital, and in all such cases only on payment, either by the patients or by the commune where they reside. The charges vary according to circumstances, ranging from about 1s. 6d. per day. There is no out-patient department or medical school connected with the hospital.

The hospital is supported partly by its own revenues, which are small, and partly by the payments of the patients, any deficit being made up by the city authorities, who largely control the administration.

